# SAMHD1 silencing cooperates with radiotherapy to enhance anti-tumor immunity through IFI16-STING pathway in lung adenocarcinoma

**DOI:** 10.1186/s12967-022-03844-3

**Published:** 2022-12-29

**Authors:** Yangyi Li, Yuke Gao, Xueping Jiang, Yajie Cheng, Jianguo Zhang, Liexi Xu, Xinyu Liu, Zhengrong Huang, Conghua Xie, Yan Gong

**Affiliations:** 1 Department of Radiation and Medical Oncology, Zhongnan Hospital of Wuhan University, Wuhan, 430071 Hubei China; 2 Department of Biological Repositories, Zhongnan Hospital of Wuhan University, Wuhan, 430071 Hubei China; 3 Hubei Key Laboratory of Tumor Biological Behaviors, Zhongnan Hospital of Wuhan University, Wuhan, 430071 Hubei China; 4 Hubei Cancer Clinical Study Center, Zhongnan Hospital of Wuhan University, Wuhan, 430071 Hubei China; 5 Tumor Precision Diagnosis and Treatment Technology and Translational Medicine, Hubei Engineering Research Center, Zhongnan Hospital of Wuhan University, Wuhan, 430071 Hubei China

**Keywords:** SAMHD1, IFI16, STING, Anti-tumor immunity, Radiotherapy, Lung adenocarcinoma

## Abstract

**Background:**

Sterile alpha motif domain and histidine-aspartate domain-containing protein 1 (SAMHD1) is a DNA end resection factor, which is involved in DNA damage repair and innate immunity. However, the role of SAMHD1 in anti-tumor immunity is still unknown. This study investigated the effects of SAMHD1 on stimulator of interferon genes (STING)-type I interferon (IFN) pathway and radiation-induced immune responses.

**Methods:**

The roles of SAMHD1 in the activation of cytosolic DNA sensing STING pathway in lung adenocarcinoma (LUAD) cells were investigated with flow cytometry, immunofluorescence, immunoblotting and qPCR. The combined effects of SAMHD1 silencing and radiation on tumor cell growth and STING pathway activation were also evaluated with colony formation and CCK8 assay. The Lewis lung cancer mouse model was used to evaluate the combined efficiency of SAMHD1 silencing and radiotherapy in vivo. Macrophage M1 polarization and cytotoxic T cell infiltration were evaluated with flow cytometry.

**Results:**

The single-stranded DNA (ssDNA) accumulated in the cytosol of SAMHD1-deficient lung adenocarcinoma (LUAD) cells, accompanied by upregulated DNA sensor IFN-γ-inducible protein 16 (IFI16) and activated STING pathway. The translocation of IFI16 from nucleus to cytosol was detected in SAMHD1-deficient cells. IFI16 and STING were acquired in the activation of STING-IFN-I pathway in SAMHD1-deficient cells. SAMHD1 silencing in LUAD cells promoted macrophage M1 polarization in vitro. SAMHD1 silencing synergized with radiation to activate ssDNA-STING-IFN-I pathway, inhibit proliferation, promote apoptosis and regulate cell cycle. SAMHD1 silencing cooperated with radiotherapy to inhibit tumor growth and increase CD86^+^MHC-II^high^ M1 proportion and CD8^+^ T cell infiltration in vivo.

**Conclusions:**

SAMHD1 deficiency induced IFN-I production through cytosolic IFI16-STING pathway in LUAD cells. Moreover, SAMHD1 downregulation and radiation cooperated to inhibit tumor growth and enhance anti-tumor immune responses through macrophage M1 polarization and CD8^+^ T cell infiltration. Combination of SAMHD1 inhibition and radiotherapy may be a potentially therapeutic strategy for LUAD patients.

**Supplementary Information:**

The online version contains supplementary material available at 10.1186/s12967-022-03844-3.

## Background

Lung cancer is one of the most common malignant tumors with the highest mortality in the world [1]. Histopathologically, approximately 50% of lung cancers are lung adenocarcinoma (LUAD) [2]. In clinical practice, most patients were already at advantage stages when they were first diagnosed with LUAD [3]. Nowadays, the clinical application of immunotherapy has greatly improved the treatment outcome of patients. However, in advanced LUAD, the response rate of programmed death-1/programmed death-ligand 1 inhibitor monotherapy is only 17–21% [4]. The optimal treatment strategy for advanced LUAD is still controversial. It is of great importance to seek combined strategies and improve tumor immune microenvironment.

Radiotherapy enhances the immunogenicity of tumor cells [5], and has a synergistic effect with immunotherapy [6]. Radioimmunotherapy generates more effective anti-tumor immune responses, but its regulatory mechanism is still being studied. Radiotherapy can active cyclic GMP-AMP synthase (cGAS)/stimulator of interferon genes (STING) signaling pathway and promote the release of pro-immune cytokines [5, 7]. Radiation directly induces DNA damage and the formation of micronuclei in cancer cells, and cause DNA single- and double-stranded breaks [8]. The accumulation of micronuclei and double-stranded DNA in the cytoplasm result in the activation of the cytoplasmic DNA sensor cGAS, which activates the STING/type I interferon (IFN) signaling pathway, promoting the infiltration of CD8^+^ T cells in tumors [9]. DNA damage repair deficiency alone or in combination with radiotherapy enhances the immunostimulatory function through IFN-I signaling pathway [7].

Sterile alpha motif domain and histidine-aspartate domain-containing protein 1 (SAMHD1) was originally identified in 2000 as an IFN-γ-inducing protein in dendritic cells [10]. In the past decade, consequent researches revealed that SAMHD1 was a key limiting factor for human immunodeficiency virus infection [11], and that its mutation caused Aicardi–Goutières syndrome, a hereditary inflammatory encephalopathy caused by excessive interferon (IFN) production [12]. Recent studies indicated that SAMHD1 formed homo tetramers in the G1 phase, playing a role as deoxy-ribonucleoside triphosphate (dNTP) hydrolase to maintain the balance of dNTP pools [13]. However, when entering the S phase, SAMHD1 was phosphorylated at T592 to promote degradation of nascent DNA at stalled replication forks and activate the ataxia-telangiectasia-mutated-and-Rad3-related kinase/checkpoint kinase 1 checkpoint. SAMHD1 deletion led to the accumulation of single-stranded DNA (ssDNA) in the cytoplasm and activate the STING signaling pathway [14]. SAMHD1 also plays important roles in DNA damage repair. It binds to Meiotic Recombination 11 (MRE11) and recruits CtBP interacting protein (CtIP) to the DNA damage sites to promote DNA end resection, activating the DNA damage repair pathway [15].

Radiotherapy causes DNA damage and activates anti-tumor immunity, and SAMHD1 participates in DNA damage repair and innate immune responses. Therefore, we supposed that the combination of SAMHD1 silencing and radiotherapy might enhance the DNA damage and augment the anti-tumor immunity. Here, we designed experiments in vitro and in vivo to investigate and verify the function of SAMHD1 in anti-tumor immunity and radiotherapy.

## Methods

### Bioinformatic analyses

Survival analysis was performed to determine the prognostic value of SAMHD1 with K-M plotter, an online database (www.kmplot.com). GSEA was performed with the GMT file (c2.KEGG.v6.2 and h.all.v7.1) gene set to download the biological processes from GSEA website (http://www.broad.nit.edu/gsea). Normalized enrichment score > 1.5 and *P* < 0.05 were defined as the significant enrichment pathway. GO and KEGG enrichment analyses were performed using the clusterProfiler package. *P* < 0.05 was considered a statistical significance.

### Cell lines and cell culture

Human LUAD cell lines, H1299, H1975, A549 and PC9 were cultured in RPMI-1640 Medium (HyClone, USA) containing 10% fetal bovine serum (Gibco, USA) in incubator (37 °C, 5% CO_2_). The Lewis lung cancer (LLC) cells and RAW264.7 cells were cultured in DMEM medium (HyClone, USA) with 10% fetal bovine serum. All cell lines were obtained from the Type Culture Center of the Chinese Academy of Sciences (Shanghai, China), and authenticated by short tandem repeat analyses.

### RNA interference, plasmid and lentiviral transfection

The transfection of small interfering RNAs (siRNAs) targeting SAMHD1, STING and IFI16 synthesized by Genepharma (Suzhou, China) was performed with jetPRIME transfection reagent (Polyplus, France). The transfection of SAMHD1 overexpression plasmid synthesized by Genechem (Shanghai, China) was performed with Lipofectamine 3000 (Thermo Fisher Scientific, USA). LLC cells were infected with short hairpin RNA-Samhd1 lentiviruses synthesized by Genechem and the stably transfected cell lines were obtained by puromycin selection (4 μg/mL). The targeting siRNA sequences were included in the supplementary file (Additional file 1: Table S1).

### RNA extraction and quantitative real-time PCR (qPCR)

The total RNA was isolated from cells using TRIzol (Vazyme, Nanjing, China). Total RNA was reversely transcripted into cDNA using hiScript Q RT Supermix with gDNA Eraser (Vazyme). SYBR Green qPCR mix (Vazyme) was used to perform qPCR in the CFX96 RT-PCR System (Bio-Rad, USA). The mRNA relative expression was calculated using 2^−ΔΔCt^ method. Primer sequences were listed in the supplementary file (Additional file 1: Table S1).

### Protein isolation and immunoblotting

The cells were broken by sonication in RIPA lysis buffer (Beyotime Biotechnology, Shanghai, China) containing phosphatase and protease inhibitors (Beyotime) to extract protein. Protein samples were boiled with 5 × loading buffer (Beyotime). SDS-PAGE gels were used to separate samples, which were then transferred to PVDF membranes. After blocking with 5% skimmed milk and incubating with primary antibodies, the bands were detected using an electrochemiluminescence detection kit (Biosharp, Beijing, China) and captured by chemiluminescence imager (Bio-Rad). The primary antibodies were included in the supplementary file (Additional file 1: Table S2).

### Immunofluorescence (IF) and immunohistochemistry (IHC)

For IF, adherent cells were fixed with 4% paraformaldehyde fixative (Biosharp) and permeated with 0.5% Triton X-100 (BioFroxx, German). The cells were then blocked with 5% bovine serum albumin (Biosharp) and then incubated with antibodies (Additional file 1: Table S2). Images were captured using a fluorescent microscope (Olympus, Japan) or the Leica STELLARIS 5 confocal microscope (Leica Microsystems, German). For IHC, after antigen retrieval and blocking endogenous peroxidase, the sections were blocked with 3% bovine serum albumin then incubated with antibodies. DAB chromogen was applied and hematoxylin counterstained nuclei. Images were acquired using a light microscope. Hematoxylin and eosin (H&E) staining was conducted to routine protocols.

### Enzyme-linked immunosorbent assay (ELISA)

Culture medium was collected from the cells. Using the mouse IFNβ ELISA kits (Bioswamp, Wuhan, China) according to the instructions, the OD values at 450 nm were determined by SpectraMax® Absorbance Reader (Molecular Devices Corporation, USA).

### Colony forming assay and CCK8 assay

After 48 h of treatments, the cells were seeded into 6-well plates (1000 cells/well) and 96-well plates (1000 cells/well). A CCK8 kit (Meilunbio, Dalian, China) was used to performed CCK8 assays. After 7–10 days of culture, the colonies were fixed with 4% paraformaldehyde, and then stained with 0.5% crystal violet (Beyotime). The numbers of colonies were then counted.

### Flow cytometry

For the investigation of ssDNA accumulation, the cells were fixed with 2% paraformaldehyde and then permeated with 0.5% Triton X-100. After that, the cells were blocked with fetal bovine serum and then incubated with the primary antibodies against ssDNA. Then the cells were incubated with secondary antibodies. Cell cycle and apoptosis were performed according instructions. The data were acquired on CytoFLEX system. To analyze CD3^+^ and CD8^+^ T cell infiltration, as well as macrophage maturation and polarization, the single cell suspensions were prepared from fresh mouse tissues. Fluorescence-labeled antibodies against CD45, CD3, CD4, CD8, CD11b, F4/80, CD86 and MHC-II were then used to stain the cells. The data were acquired on CytoFLEX system and analyzed with FlowJo V10. The antibodies were presented in the supplementary file (Additional file 1: Table S2).

### Mice and radiotherapy

To generate a subcutaneous tumor mouse model, wild-type C57BL/6 female mice (WQJX Biotechnology, Wuhan, China) aged 6–7 weeks and housed under SPF conditions were randomly divided into 4 groups using simple randomization. The sample size was decided on previous experience. Mice received injections of negtive control (NC) or shSAMHD1 stable LLC cells (5 × 10^6^ cells in 100 μl PBS) into the right armpits. Tumor volumes were determined using the following formula: (length × width^2^)/2. Mice were treated with radiotherapy 8 Gy × 3, when tumor volumes reached 500 mm^3^. Xenografts had ulcerations were excluded from the study. Mice were euthanized once tumor size reached 2000 mm^3^.The tumor volumes were detected using in vivo imaging system Spectrum 15 days from injection. All animal experiments were approved by Institutional Animal Care and Use Committee at Zhongnan Hospital of Wuhan University.

### Statistical analysis

This study used GraphPad Prism to process all the data. Quantitative results were expressed as the mean ± standard deviation. The student’s t-test was used to compare the difference between 2 groups and one-way ANOVA was used to compare 3 or more groups. Survival rates were calculated by the Kaplan–Meier (KM) plots and compared using log-rank tests. *P* < 0.05 was considered statistically significant.

## Results

### SAMHD1 silencing caused cytosolic ssDNA accumulation in LUAD

**Fig. 1:**
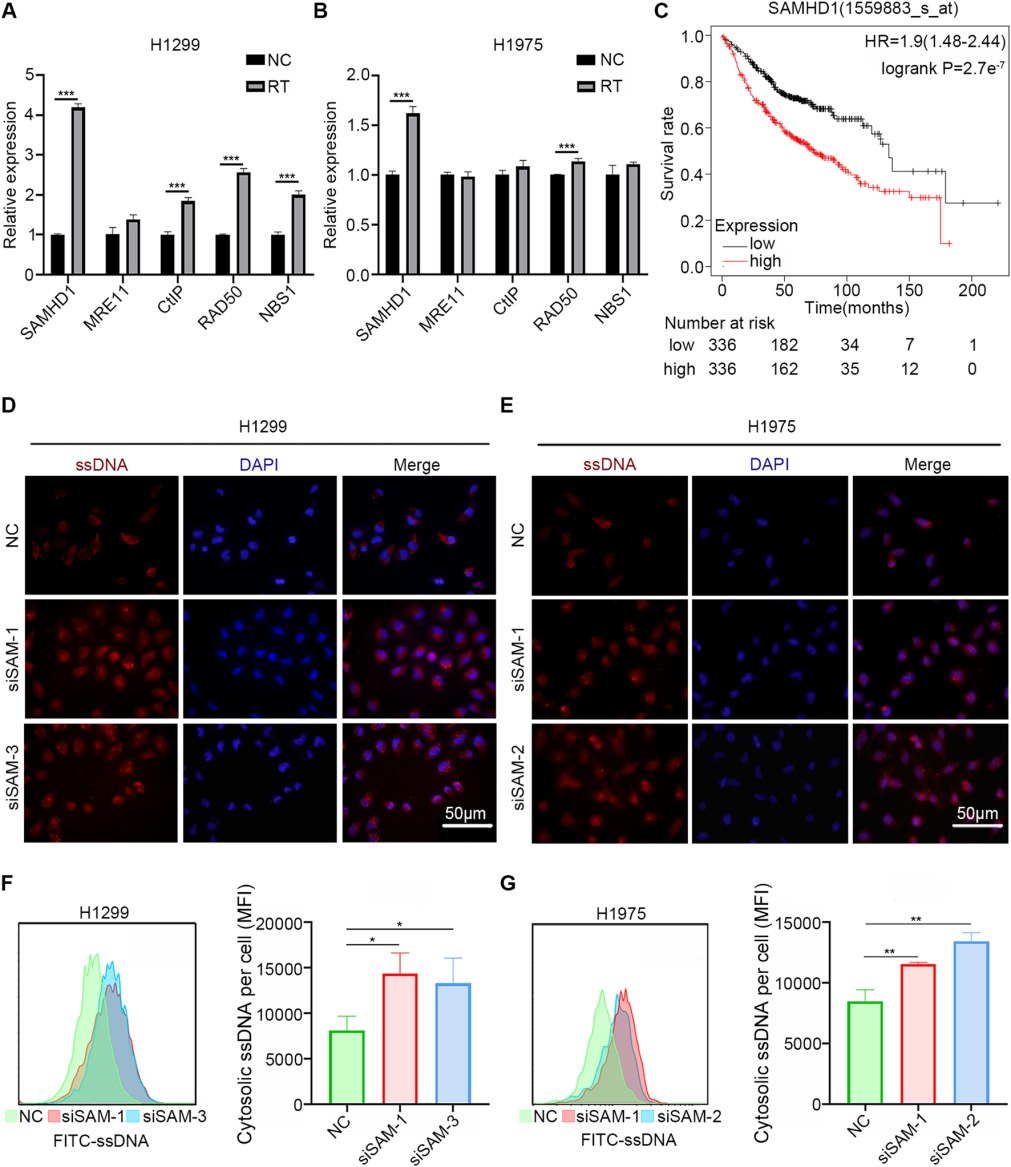
SAMHD1 silencing caused cytosolic ssDNA accumulation in LUAD cells. **A, B** The mRNA levels of DNA end resection factors: SAMHD1, MRE11, CtIP, RAD50, NBS1 in H1299and H1975 cells with radiation were detected with qPCR. **C** Overall survival analysis of LUAD was performed using KM plotter. **D, E** Immunofluorescence was performed to detect the accumulation of ssDNA in the cytoplasm of H1299 and H1975 cells. **F, G** The accumulation of ssDNA in cytoplasm was detected by flow cytometry in H1299 and H1975 cells. The cytosolic ssDNA was evaluated by mean fluorescence intensity (MFI). Scale bar: 50 μm. N = 3; *, *P* < 0.05, **, *P* < 0.01, ***, *P* < 0.001

DNA end resection has a critical role in the initiation of double strand breaks ends for efficient homologous recombination repair, regulating DNA damage repair and cell radiosensitivity [16–19]. Previous studies suggested that DNA end resection factors regulated the generation of ssDNA fragments and initiation of innate immune responses [20, 21]. Thus, these factors might increase the tolerant of tumor cells to radiation and participate in the radiation-induced immune responses. We detected the expression levels of several DNA end resection factors, SAMHD1, MRE11, CtIP, and double strand break repair protein RAD50, Nijmegen breakage syndrome 1 (NBS1) in LUAD cells upon radiation. The qPCR results showed that SAMHD1 mRNA levels were significantly increased (Fig. 1A, B). The protein levels of SAMHD1 were also upregulated upon radiation in LUAD cells (Additional file 1: Fig. S1A).

The prognostic value of SAMHD1 expression on LUAD was investigated using K-M plotter. High SAMHD1 expression was correlated with poor prognosis (Fig. 1C). To explore the biological function of SAMHD1, differentially expressed genes in high and low SAMHD1 expressing groups were integrated into GO and KEGG analysis, and the results showed that SAMHD1 was associated with biological processes, leukocyte proliferation, leukocyte migration and myeloid leukocyte migration, which were immunity-related functions (Additional file 1: Fig. S1B). The GSEA was used to analyze the signaling pathways related to SAMHD1. In the SAMHD1 high-expression groups, genes were enriched in cancers, cytosolic DNA sensing, chemokine signaling, cytokine-cytokine receptor interaction, apoptosis and antigen processing and presentation pathway (Additional file 1: Fig. S1C).

We detected the expression levels of SAMHD1 in several LUAD cell lines. The results showed that SAMHD1 expression levels in H1299 and H1975 cells were higher than those in A549 and PC9 cells (Additional file 1: Fig. S2A, B). Therefore, we chose H1299 and H1975 cells for SAMHD1 knockdown assays. Flow cytometry and IF were used to detect cytosolic ssDNA in siSAMHD1 LUAD cells. SAMHD1 silencing significantly induced the accumulation of cytosolic ssDNA in H1299 and H1975 cells (Fig. 1D, E). Flow cytometry analysis (Fig. 1F, G) were consistent with the IF results.

### SAMHD1 silencing activated IFI16 and TBK1-IRF3-IFN-I pathway in LUAD

**Fig. 2:**
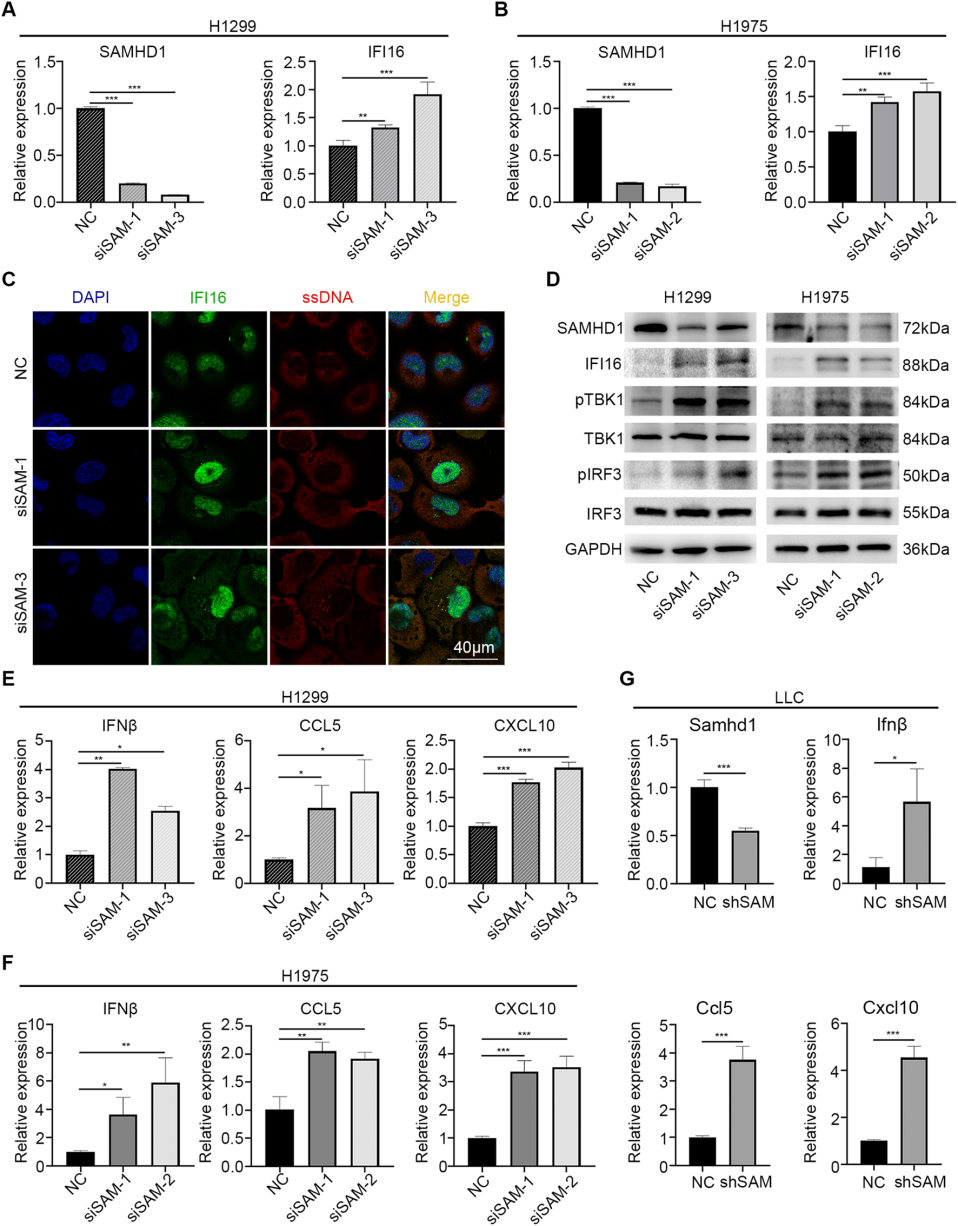
SAMHD1 silencing activated IFI16 and TBK1-IRF3-IFN-I pathway in LUAD cells. **A**, **B** The mRNA levels of SAMHD1 and IFI16 were detected by qPCR in H1299 and H1975 cells. **C** The translocation of IFI16 from nucleus to cytosol in H1299 cells was detected with confocal. **D** IFI16 and TBK1-IRF3 pathway protein levels in H1299 and H1975 cells were detected by immunoblotting. **E**, **F** The mRNA levels of IFNβ, CCL5 and CXCL10 in H1299 and H1975 cells were detected by qPCR. **G** The mRNA levels of Ifnβ, Ccl5 and Cxcl10 in LLC cells were detected by qPCR. Scale bar: 40 μm. N = 3, *, *P* < 0.05; **, *P* < 0.01; ***, *P* < 0.001

Since IFI16 is a key DNA sensor, which senses ssDNA and double-stranded DNA [22], we tested whether the ssDNA fragments induced by SAMHD1 silencing could activate IFI16. The mRNA levels of IFI16 were increased in the siSAMHD1 cells (Fig. 2A, B). Confocal images also confirmed the translocation of IFI16 from the nuclear to the cytoplasm (Fig. 2C), indicating the activation of IFI16 [22, 23]. Immunoblotting showed that SAMHD1 deficiency resulted in increased phosphorylation of IFN regulatory factor (IRF) 3 and TANK-binding kinase (TBK) 1 (Fig. 2D), which mediated innate immune sensing with IFN-I production [24]. The results of qPCR revealed that SAMHD1 deficiency increased the mRNA levels of IFNβ, CCL5 and CXCL10 (Fig. 2E, F), which were the key IFN-I-related immune molecules. Meanwhile, SAMHD1 overexpression decreased IFNβ, CCL5 and CXCL10 production (Additional file 1: Fig. S2C, D), and inhibit phosphorylation of TBK1 and IRF3 (Additional file 1: Fig. S2E). We also confirmed the activation of TBK1-IRF3-IFN-I signaling pathway in shSAMHD1 LLC cells (Fig. 2G, Additional file 1: Fig. S2F, G).

### SAMHD1 silencing inhibited tumor growth and promoted macrophage M1 polarization

**Fig. 3:**
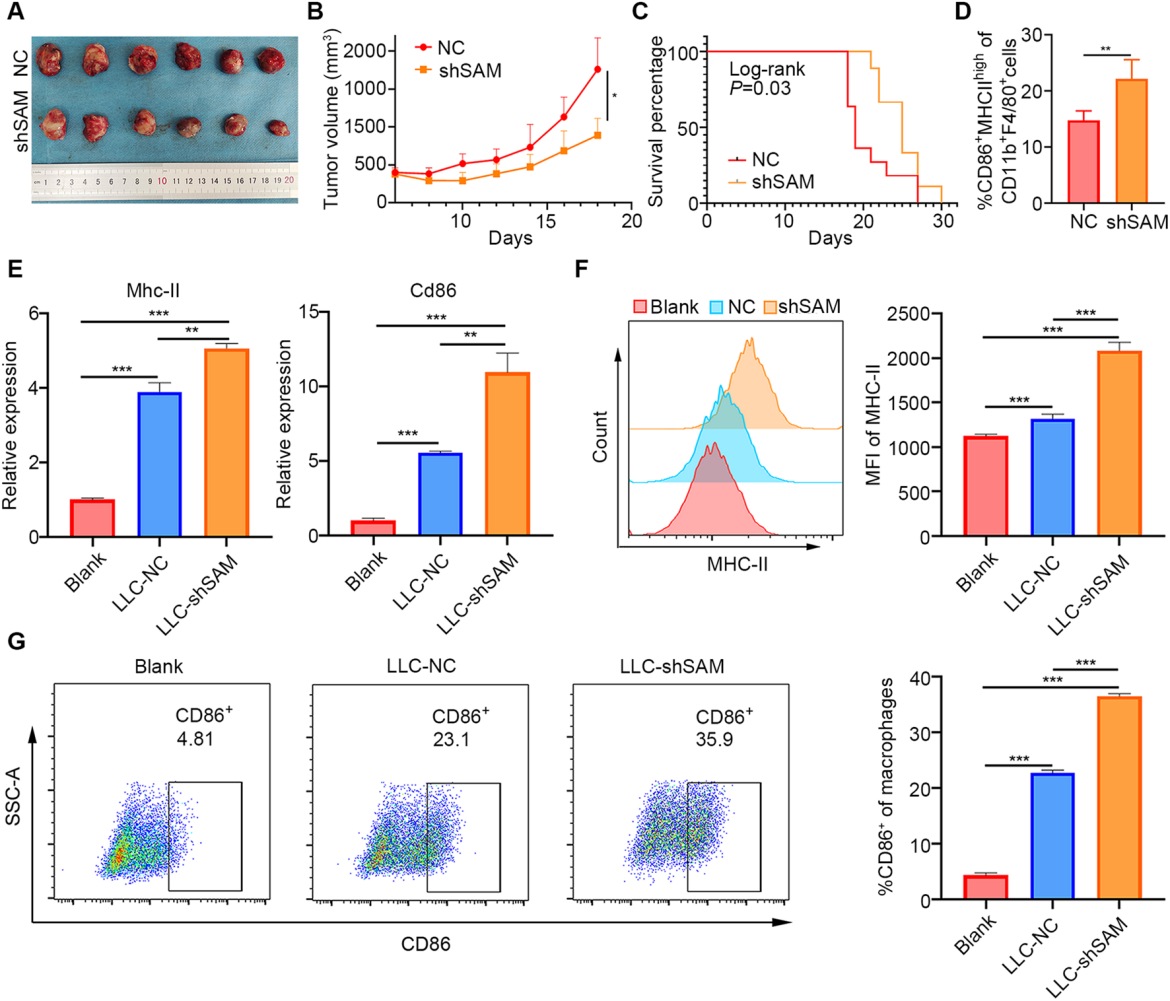
SAMHD1 silencing inhibited tumor growth and promoted macrophage M1 polarization. **A** C57BL6 mice were injected with LV-NC and LV-shSAMHD1 infected LLC cells. On the 20th day of injection, the tumors were collected for photograph. **B** The tumor volumes were measured every 2 days and depicted in the line chart, N ≥ 8. **C** Mice survival was recorded for KM curves, N ≥ 9. **D** Quantitative analysis of MHC-II^+^CD86^+^ macrophages in tumors, N ≥ 4. **E** The M1 related molecules Mhc-II and Cd86 mRNA expression in co-cultured RAW264.7 cells were detected by qPCR. **F** The surface expression of MHC-II was detected by flow cytometry and analyzed by MFI. **G** Representative flow cytometry of CD86^+^ cells. Quantitative analysis of CD86^+^ macrophages. N = 3; *, *P* < 0.05; **, *P* < 0.01; ***, *P* < 0.001

To test the anti-tumor effects in vivo, C57BL/6 mice were injected with LV-shSAMHD1 and LV-NC LLC cells. The tumor volumes of shSAMHD1 group were lower than the NC group (Fig. 3A). SAMHD1 silencing significantly suppressed tumor growth in vivo (Fig. 3B). In addition, the shSAMHD1 group showed prolonged survival compared to the NC group (Fig. 3C). CD86 and major histocompatibility complex (MHC)-II were highly expressed on the surface of M1 macrophages. The percentage of CD86^+^MHC-II^high^ cells were higher in the shSAMHD1 group (Fig. 3D). Then we investigated whether SAMHD1 silencing could affect macrophage M1 polarization in vitro. The medium of SAMHD1-deficient LLC cells was collected to culture RAW 264.7 cells. The mRNA levels of Mhc-II, Cd86 were increased (Fig. 3E). SAMHD1 silencing in LLC cells increased the expression of MHC-II (Fig. 3F) and the percentage of CD86^+^ macrophages (Fig. 3G).

### SAMHD1 regulation of TBK1-IRF3-IFN-I pathway is IFI16 and STING dependent

**Fig. 4:**
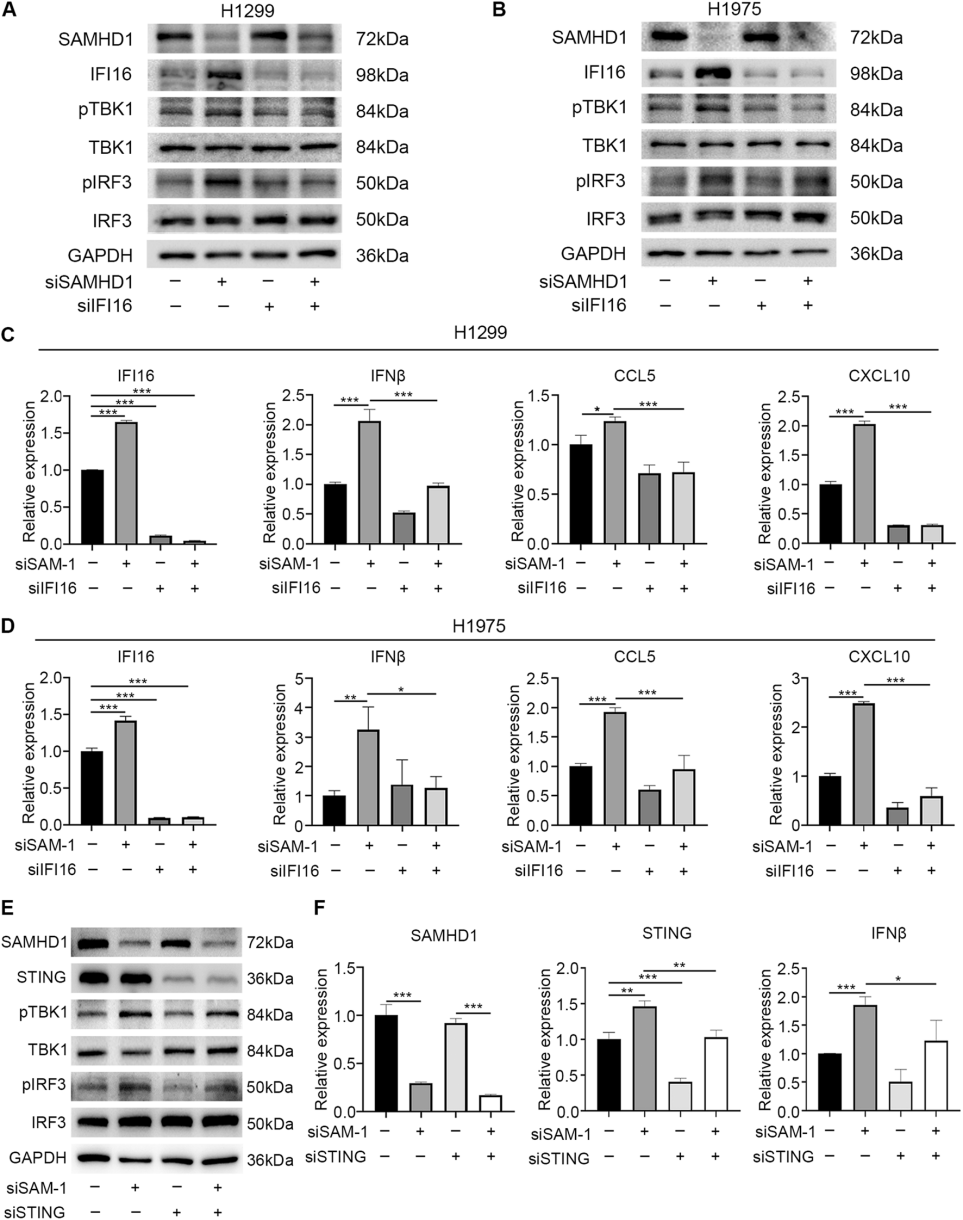
SAMHD1 regulation of TBK1-IRF3-IFN-I pathway was IFI16 and STING dependent. **A**, **B** The effects of siSAMHD1, siIFI16 or their combination on TBK1-IRF3 pathway in H1299 and H1975 cells were evaluated by immunoblotting. **C**, **D** The mRNA levels of IFI16, IFNβ, CCL5 and CXCL10 were detected by qPCR after transfecting siSAMHD1, siIFI16 or combination in H1299 and H1975 cells. **E** The effects of siSAMHD1, siSTING or their combination on TBK1-IRF3 pathway in H1299 cells were evaluated by immunoblotting. **F** The mRNA levels of SAMHD1, STING and IFNβ in H1299 cells were detected by qPCR after transfecting siSAMHD1, siSTING or combination. N = 3; *, *P* < 0.05; **, *P* < 0.01; ***, *P* < 0.001

Since SAMHD1 silencing caused ssDNA accumulation, which then activated the DNA sensor IFI16, we assumed that IFI16 was essential for the activation of STING-IFN-I pathway. IFI16 knockdown abrogated the phosphorylation of TBK1 and IRF3 in response to SAMHD1 silencing (Fig. 4A, B). The results of qPCR confirmed that IFI16 knockdown inhibited the production of IFNβ, CCL5 and CXCL10 induced by SAMHD1 deficiency (Fig. 4C, D). These results indicated that IFI16 was required in the SAMHD1 regulation of TBK1-IRF3-IFN-I pathway.

The cytosolic DNA sensing STING pathway was required in the IFN-I response in SAMHD1-deficient mice [25] and cancer cells [14]. To test whether STING results IFN-I production in SAMHD1-silencing LUAD cells, STING was downregulated by siRNA. SAMHD1 deficiency increased the phosphorylation of TBK1 and IRF3, and STING silencing downregulated their phosphorylation (Fig. 4E). SAMHD1 deficiency upregulated STING downstream cytokines IFNβ, and this effect was partially inhibited by STING knockdown (Fig. 4F). These results suggested that STING mediated the regulation of IFN-I response by SAMHD1 in LUAD cells.

### SAMHD1 silencing collaborated with radiation to induce ssDNA accumulation, activate TBK1-IRF3-IFN-I signaling and inhibit LUAD cells growth

**Fig. 5:**
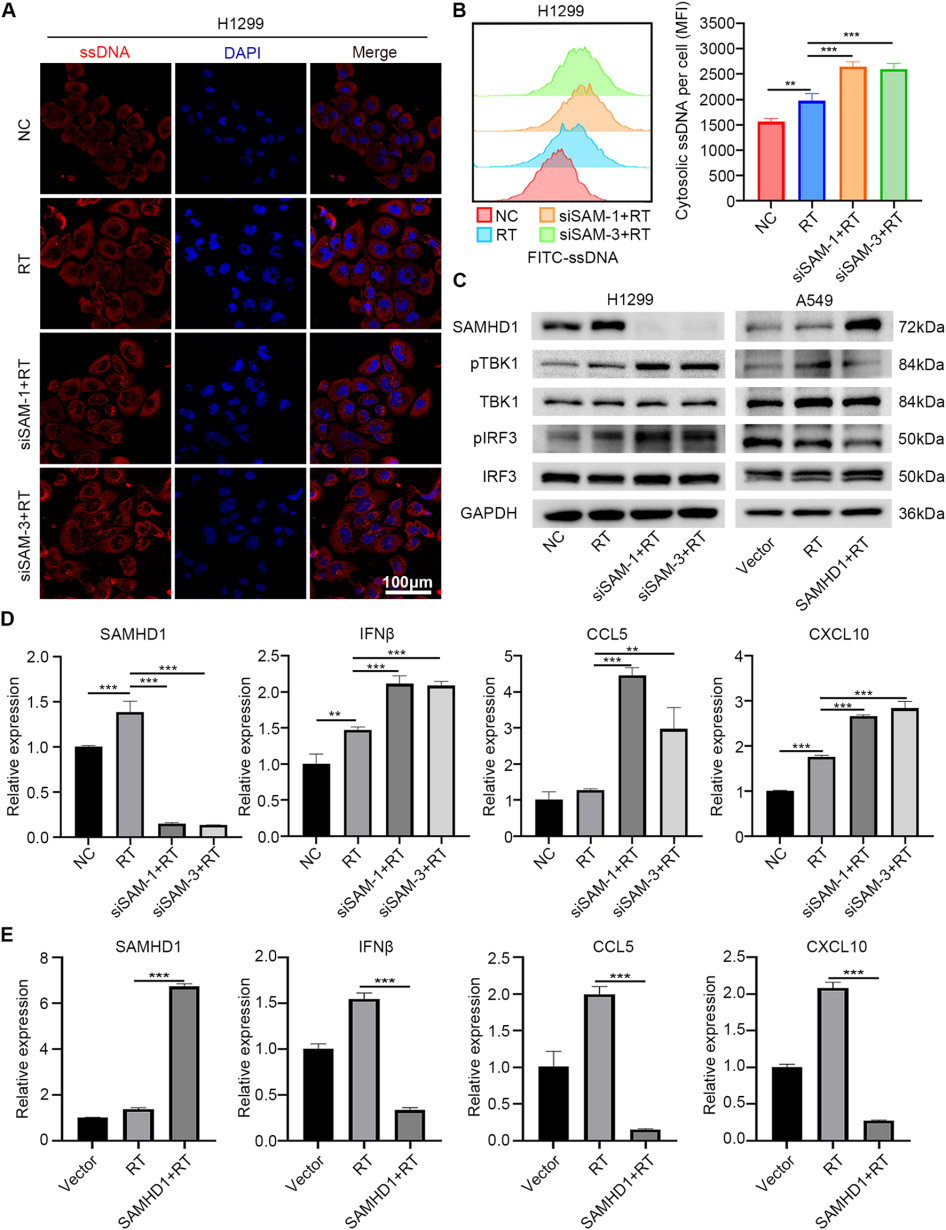
SAMHD1 silencing collaborated with radiotherapy to induce ssDNA accumulation and activate TBK1-IRF3-IFN-I signaling. **A** Confocal images were performed to detect the accumulation of ssDNA in cytoplasm in H1299 cells after radiation and transfecting siSAMHD1. **B** The accumulation of ssDNA in cytoplasm after radiation and siSAMHD1 transfection was detected by flow cytometry. The cytosolic ssDNA was evaluated by MFI. **C** TBK1-IRF3 pathway protein levels in H1299 and A549 cells after transfection and radiation were detected by immunoblotting. **D** The mRNA levels of SAMHD1, IFNβ, CCL5 and CXCL10 after SAMHD1 silencing and radiation in H1299 cells were detected by qPCR. **E** The mRNA levels of SAMHD1, IFNβ, CCL5 and CXCL10 after SAMHD1 overexpression and radiation in A549 cells were detected by qPCR. N = 3; *, *P* < 0.05; **, *P* < 0.01; ***, *P* < 0.001

Since SAMHD1 plays an important role in DNA end resection, which is the initiation of DNA damage repair signaling pathway [15], we hypothesized that SAMHD1 silencing and radiation had synergistic effects in activating cytosolic DNA sensing TBK1-IRF3-IFN-I signaling pathway. Confocal images and flow cytometry showed that SAMHD1 silencing and radiation synergistically increased ssDNA accumulation in cytosol (Fig. 5A, B, Additional file 1: Fig. S3A, B).

H1299 and A549 cells were treated with siSAMHD1 and/or radiation. A549 and PC9 cells were treated with SAMHD1 overexpression and/or radiation. As expected, SAMHD1 silencing enhanced radiation-induced TBK1 and IRF3 phosphorylation, and SAMHD1 overexpression inhibited radiation-induced TBK1 and IRF3 phosphorylation (Fig. 5C, Additional file 1: Fig. S3C). Consistently, SAMHD1 silencing and radiation further increased the mRNA level of IFNβ, CCL5, CXCL10 (Fig. 5D, Additional file 1: Fig. S3D). SAMHD1 overexpression inhibited radiation-induced IFNβ, CCL5, CXCL10 production (Fig. 5E, Additional file 1: Fig. S3E).

**Fig. 6:**
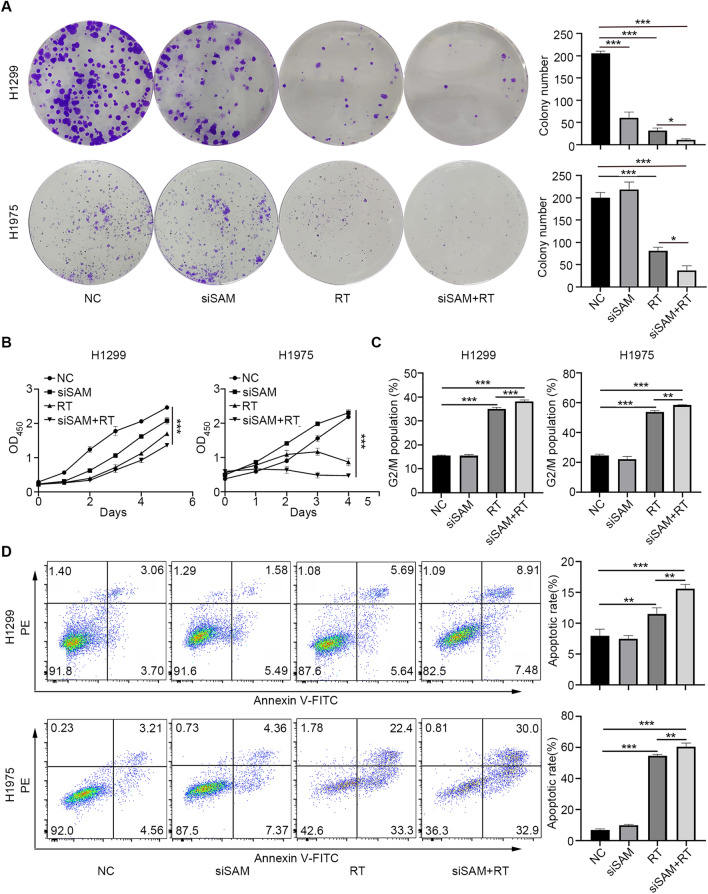
SAMHD1 silencing collaborated with radiation to inhibit LUAD cells growth. **A** The effects of SAMHD1 silencing and radiation on the colony formation in H1299 and H1975 cells. Colony growth was quantified as colony number. **B** CCK8 assays were performed to evaluate the cell growth inhibition of siSAMHD1 and radiation. **C** The G2/M phase population were analyzed by flow cytometry after transfection and radiation. **D** The effects of siSAMHD1 and radiation on apoptosis were detected using flow cytometry. The statistical analyses on apoptosis rates. N = 3; *, *P* < 0.05; **, *P* < 0.01; ***, *P* < 0.001

We also examined the synergistic anti-tumor effects of SAMHD1 silencing and radiation. The colony formation and CCK8 assay showed that SAMHD1 silencing enhanced the inhibition of cell proliferation induced by radiation (Fig. 6A, B). Flow cytometry was used to detect the difference of cell cycle between each group. Radiation caused increased G2/M phase population while the combination led to an even greater increase (Fig. 6C). Implications of radiation and SAMHD1 silencing in apoptosis were also detected by flow cytometry. SAMHD1 knockdown collaborated with radiation to enhance apoptosis (Fig. 6D). Consistently, SAMHD1 overexpression alleviated radiation-induced inhibition of cell proliferation (Additional file 1: Figure. S4A, B) and radiation-induced apoptosis (Additional file 1: Figure. S4C, D). These results indicated that SAMHD1 silencing and radiation cooperated on inhibition of tumor growth and STING-IFN-I signaling pathway activation.

### SAMHD1 silencing synergized with radiotherapy to inhibit tumor growth and increase macrophage M1 polarization and CD8^+^ T cell infiltration

**Fig. 7:**
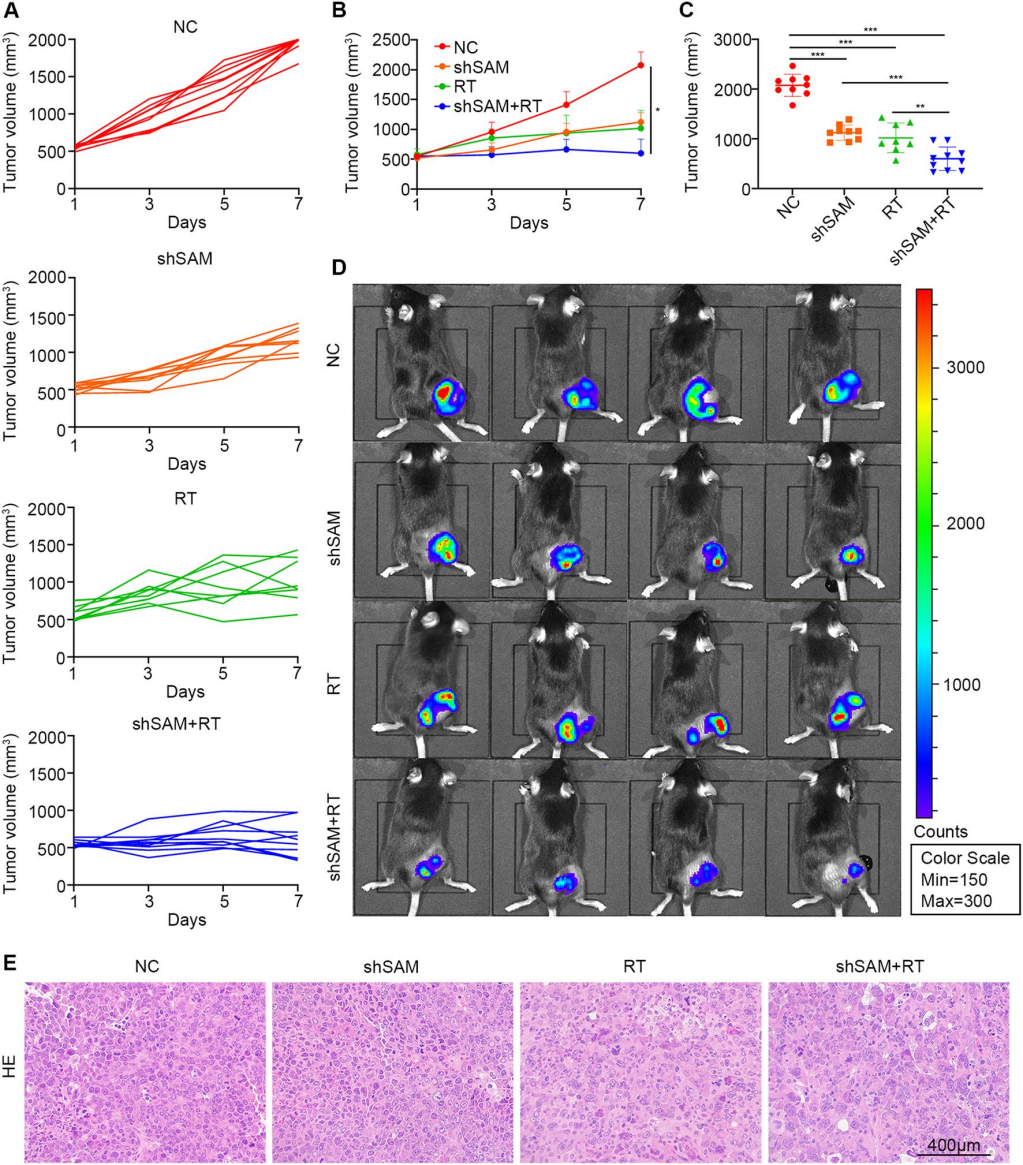
SAMHD1 silencing synergized with radiotherapy to inhibit tumor growth in vivo. **A** The mice were treated with radiotherapy when the tumor volumes were about 500 mm^3^. Tumor growth curves of individual mice in different groups. **B** LLC tumor growth curves. **C** The tumor volume on the 7th day from tumor volume reached 500 mm^3^. **D** IVIS spectrum imaging of tumor-bearing mice. **E** Representative H&E staining of tumor tissues. Scale bar: 400 μm. N ≥ 8; *, *P* < 0.05; **, *P* < 0.01; ***, *P* < 0.001

SAMHD1 silencing synergized with radiotherapy to inhibit LUAD cell growth in vitro, so we examined the potential role of SAMHD1 in tumor growth in vivo. LV-shSAMHD1 and LV-NC LLC cells were implanted into C57BL/6 mice. Radiotherapy or SAMHD1 silencing could inhibit tumor growth alone, but the combination of them would slow tumor growth more significantly (Fig. 7A, B). IVIS Spectrum and the calculated tumor volumes showed that the tumor size in the combination group were lowest (Fig. 7C, D). H&E staining indicated that tumor cell density was reduced in the radiotherapy group and the combination group (Fig. 7E).

**Fig. 8:**
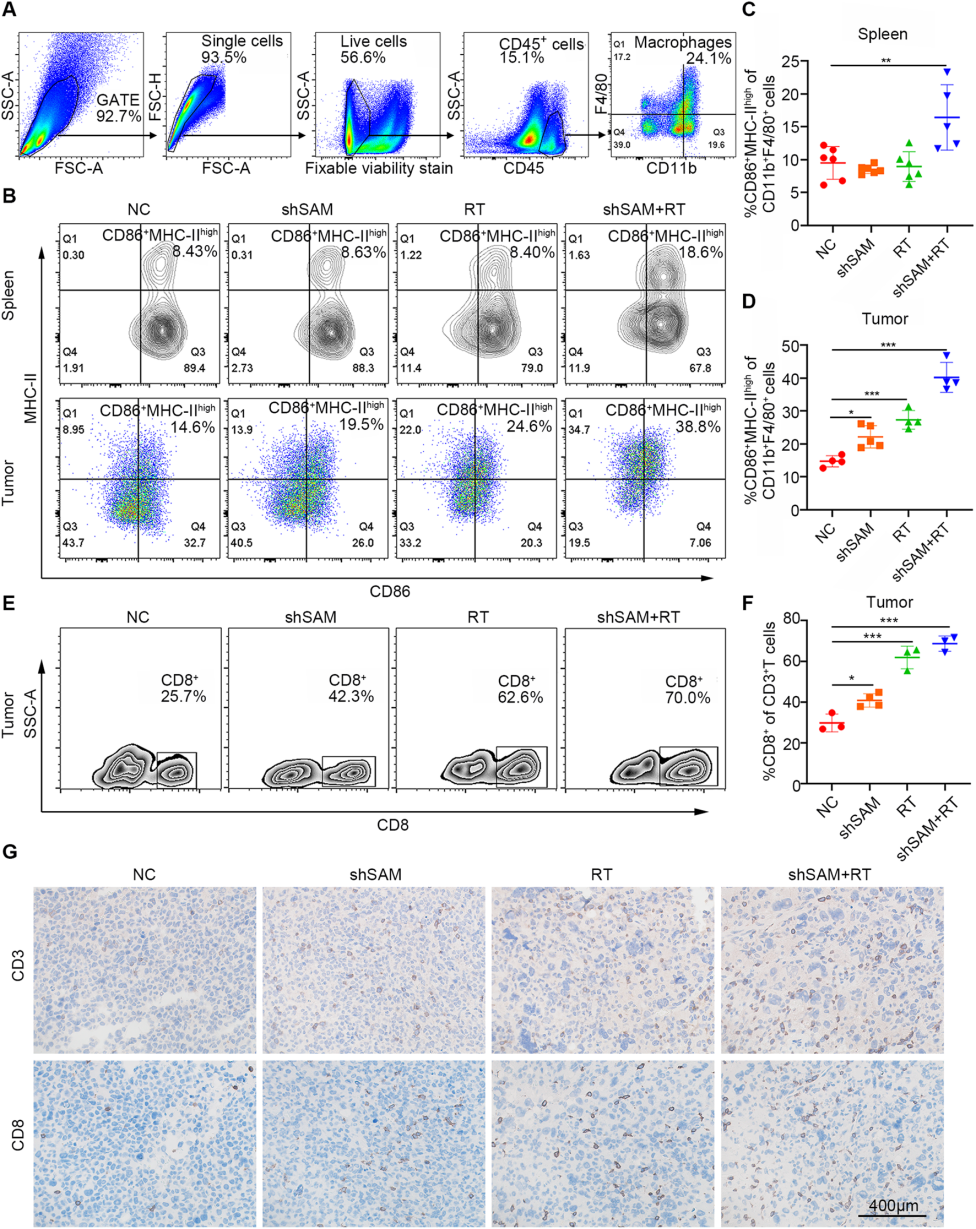
SAMHD1 silencing synergized with radiotherapy to increase macrophage M1 polarization and CD8^+^ T cell infiltration. **A** The flow cytometry gate strategy of macrophages. **B** Representative flow cytometry of MHC-II^high^CD86^+^ macrophages in spleens and tumors. **C**, **D** Quantitative analysis of MHC-II^high^CD86^+^ macrophages in spleens and tumors. **E**, **F** Representative flow cytometry of CD8^+^ T cells in tumors. Quantitative analysis of CD8^+^ T cells in tumors. **G** Representative IHC staining (CD3 and CD8) of tumor tissues. Scale bar: 400 μm. N ≥ 3; *, *P* < 0.05; **, *P* < 0.01; ***, *P* < 0.001

We hypothesized that SAMHD1 silencing and radiotherapy cooperated to inhibit tumor growth via altering tumor microenvironment. Flow cytometry was used to examine macrophage polarization and cytotoxic T cell proportion in spleens and tumor tissues. Combined therapy significantly increased the proportion of M1 macrophages in spleens. In tumors, both SAMHD1 silencing and radiation resulted in an increase of M1 macrophages, while the combination led to an even greater increase (Fig. 8A–D). In the spleen, SAMHD1 silencing and the radiotherapy increased the ratio of CD3^+^ and CD8^+^ T cells, but radiotherapy alone did not significantly affect the proportion of splenic lymphocytes (Additional file 1: Fig. S5A-D). In the tumor microenvironment, both SAMHD1 silencing and radiotherapy increased the ratio of CD8^+^ T cells in the tumor tissues, while the combined therapy had more obvious effects (Fig. 8E, F). IHC was used to analyze the ratio of CD3^+^ and CD8^+^ T cells in tumor tissues. The results showed that SAMHD1 silencing and radiotherapy increased the proportion of CD3^+^ and CD8^+^ T cells (Fig. 8G).

## Discussion

SAMHD1 (72 kDa, 626aa) is located at human chromosome 20q11.23 as a dNTP hydrolase [26] and a DNA end resection factor [27], which was involved in DNA damage repair and innate immune responses [28]. In recent years, SAMHD1 mutations were reported in several cancers, such as colorectal cancer, breast cancer and chronic lymphocytic leukemia [29]. The functional consequences of SAMHD1 in cancer development and treatment still require further researches. In the colorectal cancer, high expression SAMHD1 correlated with metastasis [30] and indicated poor prognosis of stage II patients [31]. Consistent with our results, Eudald Felip et al. found that low expression of SAMHD1 was associated with a positive prognosis in breast, ovarian and non-small cell lung cancer patients [32].

SAMHD1 was reported to suppress innate immune responses in human monocytic cells and macrophages via inhibiting interferon pathways [33]. The IFN-I responses in SAMHD1-deficient myeloid cells required the cGAS-STING cytosolic DNA sensing pathway [25]. Another study showed that SAMHD1 deficiency led to ssDNA accumulate in the cytosol and activated the cGAS-STING pathway to induce IFN-I [14]. Since cGAS is a DNA sensor which preferentially binds to double-stranded DNA [34], we wondered how ssDNA activated STING pathway. Ahmed Emam et al. found that the increased cytosolic ssDNA contains ribosomal DNA that can bind to cGAS and activate of the innate immune response [35]. Kiwon Park et al. found that SAMHD1 prevents R-loop formation to preserve genome integrity [36]. R-loops, nucleic acid structures containing RNA: DNA hybrids and ssDNAs, could be recognized by cGAS and activate cGAS-STING activity [37]. Here, we suggested SAMHD1 silencing activated STING pathway through IFI16. IFI16 is a key DNA sensor which could sense ssDNA. The non-canonical IFI16/STING pathway was reported in recent year [38]. IFI16 could promote production and function of cGAMP [39] and cooperate with cGAS in the activation of STING [23]. Our studies suggested that SAMHD1 silencing in LUAD cells caused cytosolic ssDNA accumulation and IFI16 was upregulated and translocated from nucleus to cytosol and then activated STING-IFN-I signaling pathway.

Macrophage constitutes a predominant component of tumor immune microenvironment in lung cancer [40]. The activation states of macrophages are complex. There are two main macrophage phenotypes, proinflammatory (M1) and anti-inflammatory (M2) macrophages [41]. M1 macrophages can directly mediate cytotoxicity to kill tumor cells or kill tumor cells by antibody-dependent cell-mediated cytotoxicity [42]. M1 macrophages can also enhance antigen processing and presentation and T cell responses [43]. We found that SAMHD1 silencing in lung cancer cells promoted macrophage M1 polarization, which might improve the tumor immune microenvironment.

SAMHD1 promotes DNA end resection which is the initiation of DNA repair by homologous recombination [15]. SAMHD1 silencing causes homologous recombination deficiency which may sensitize tumor cells to radiotherapy. We verified the combination effects of SAMHD1silencing and radiotherapy on tumor growth inhibition and anti-tumor immunity activation. The combination treatment inhibited cell proliferation, regulated cell cycle and increased apoptosis. In vivo, SAMHD1 deficiency and radiotherapy cooperated to inhibit tumor growth and increased M1 macrophages and CD8^+^ T cell infiltration.

There is one therapeutic implication of our findings that SAMHD1 inhibition and radiotherapy may be a rational combination to inhibit tumor growth and enhance anti-tumor immunity. The Vpx protein could induce the degradation of SAMHD1 [44]. TRIM21 is an E3 ubiqutin ligase, a key regulator of SAMHD1 which specifically degrades SAMHD1 through the proteasomal pathway [45]. But a protein-based therapy have some limitations and they also have other targets. Selective CDK4/6 inhibitors could control SAMHD1 function by inhibiting its phosphorylation [46]. CDK4/6 inhibitors might inhibit the DNA end resection ability of SAMHD1 and enhance the dNTP hydrolase function, since SAMHD1 formed homotetramers to Hydrolyze dNTP and was phosphorylated to promote DNA end resection [28]. We supposed that CDK4/6 inhibitors and radiotherapy might be a promising therapeutic combination for cancer therapy to enhance anti-tumor immune responses. Another potential therapeutic implication is that LUAD with low SAMHD1 expression might receive more benefits and immunostimulatory effects from radiotherapy.

## Conclusions

**Fig. 9:**
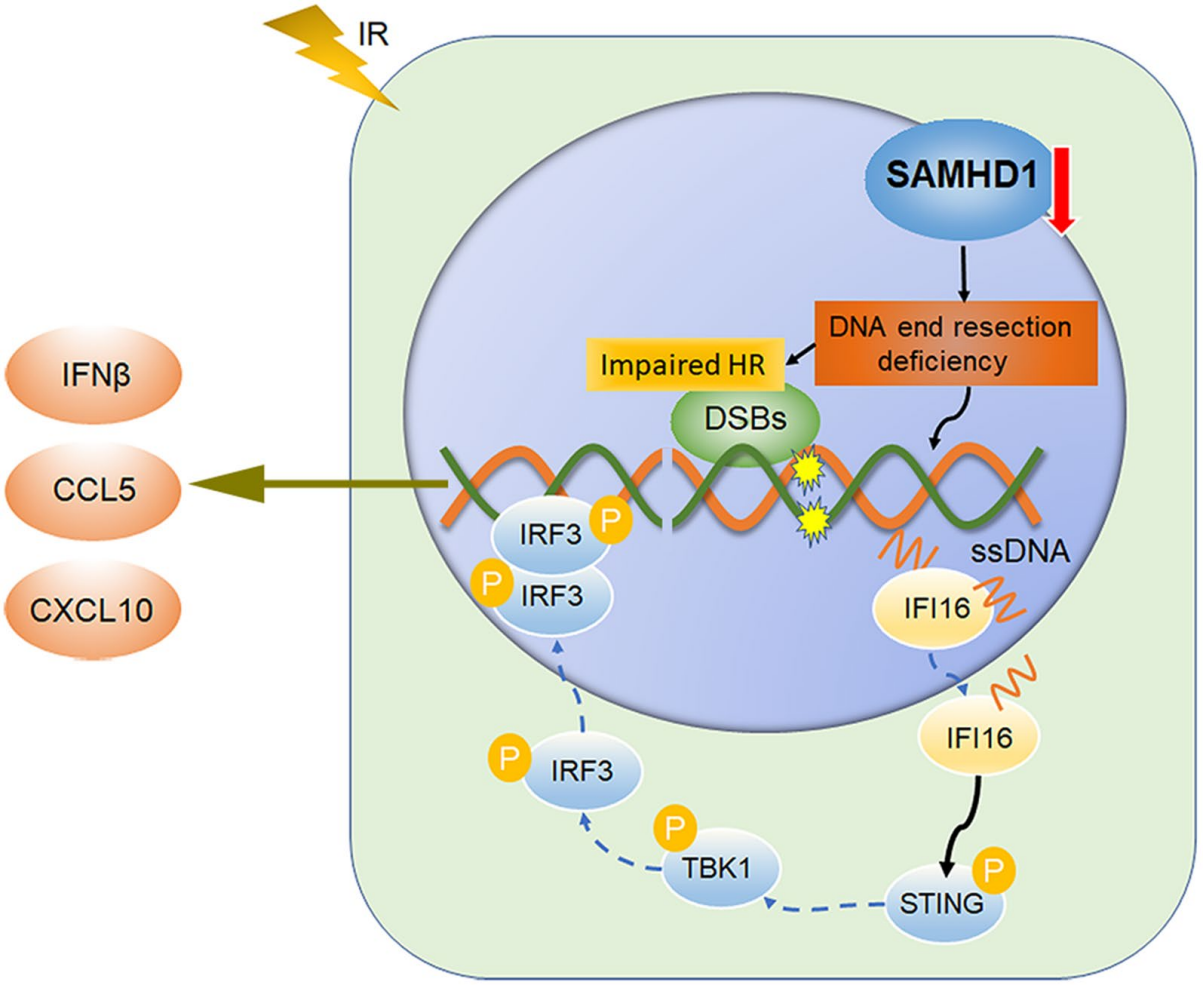
The combination of SAMHD1 silencing and radiotherapy promotes STING-IFN-I pathway activation in LUAD cells. SAMHD1 knockdown causes DNA end resection deficiency which impairs HR repair. SAMHD1 silencing collaborates with radiotherapy to induce ssDNA accumulation. The ssDNA fragments activate cytosolic DNA sensor IFI16. Then IFI16 activates STING. This triggers the phosphorylation of TBK1 and then the phosphorylation of IRF3. Thereby the p-IRF3 forms IRF3 homodimers that translocate into nucleus and results in the increased expression of IFN-I, including IFNβ, CCL5 and CXCL10

SAMHD1 deficiency induced IFN-I production through the cytosolic DNA sensing IFI16-STING signaling pathway in LUAD cells. The combination of SAMHD1 silencing and radiation enhanced the activation of TBK1-IRF3-IFN-I signaling pathway in LUAD cells (Fig. 9). In addition, SAMHD1 knockdown combined with radiotherapy inhibited tumor growth and induced anti-tumor immunity via promoting macrophage M1 polarization and CD8^+^ T cell infiltration.

## Supplementary Information

**Additional file 1: Table S1.** Primer sequences used for amplification and the targeting siRNA sequences. **Table S2.** Antibodies used in this study. **Figure S1.** Functional enrichment analysis to confirm the association of SAMHD1 with immunity. **Figure S2.** SAMHD1 inhibited TBK1-IRF3-IFN-I pathway in LUAD cells. **Figure S3.** SAMHD1 silencing synergized with radiotherapy to induce ssDNA accumulation and activate TBK1-IRF3-IFN-I signaling. **Figure S4.** SAMHD1 overexpression alleviated radiation-induced inhibition of LUAD cells growth. **Figure S5.** SAMHD1 silencing synergized with radiotherapy to regulate CD3^+^ and CD8^+^ T cell infiltration in spleen.

## Data Availability

The original contributions presented in the study are included in the article/additional file.

## Abbreviations

LUAD: Lung adenocarcinoma
SAMHD1: Sterile alpha motif domain and histidine-aspartate domain-containing protein 1
STING: Stimulator of interferon genes
IFI16: Interferon-γ-inducible protein 16
cGAS: Cyclic GMP-AMP synthase
IFN-I: Type I interferon
dNTP: Deoxy-ribonucleoside triphosphate
ssDNA: Single-stranded DNA
MRE11: Meiotic recombination 11
CtIP: CtBP interacting protein
NBS1: Nijmegen breakage syndrome 1
GSEA: Gene set enrichment analysis
qPCR: Quantitative real-time PCR
siRNA: Small interfering RNA
LLC: Lewis lung cancer cells
TBK1: TANK binding kinase 1
IRF3: Interferon regulatory factor 3
LV: Lentiviruses
shRNA: Short hairpin RNA
NC: Negative control
ELISA: Enzyme linked immunosorbent assay
MFI: Mean fluorescence intensity
MHC-II: Major histocompatibility complex II
IF: Immunofluorescence
IHC: Immunohistochemistry
H&E: Hematoxylin and eosin
