# Supplementary material for: SAMHD1 silencing cooperates with radiotherapy to enhance anti-tumor immunity through IFI16-STING pathway in lung adenocarcinoma
Source: J Transl Med. 2022 Dec 29;20:628. doi: 10.1186/s12967-022-03844-3 (PMC9798699; doi:10.1186/s12967-022-03844-3)
Supplement: Supplementary file 1 — Additional file 1: Table S1. Primer sequences used for amplification and the targeting siRNA sequences. Table S2. Antibodies used in this study. Figure S1. Functional enrichment analysis to confirm the association of SAMHD1 with immunity. Figure S2. SAMHD1 inhibited TBK1-IRF3-IFN-I pathway in LUAD cells. Figure S3. SAMHD1 silencing synergized with radiotherapy to induce ssDNA accumulation and activate TBK1-IRF3-IFN-I signaling. Figure S4. SAMHD1 overexpression alleviated radiation-induced inhibition of LUAD cells growth. Figure S5. SAMHD1 silencing synergized with radiotherapy to regulate CD3+ and CD8+ T cell infiltration in spleen. [file 12967_2022_3844_MOESM1_ESM.pdf]

Additional Tables

Table S1. Primer sequences used for amplification and the targeting siRNA sequences.

Gene	Sequence
GAPDH Fp	ACAACTTTGGTATCGTGGAAGG
GAPDH Rp	GCCATCACGCCACAGTTTC
SAMHD1 Fp	CCCAAAGTATTGCTAGACGTGA
SAMHD1 Rp	TGCATTCCATAATCCATGTTG
MRE11 Fp	ATCGGCCTGTCCAGTTTGAAA
MRE11 Rp	TGCCATCTTGATAGTTCACCCAT
CtIP Fp	AGCGTTTGTGGAGCCGTAT
CtIP Rp	GTTGGGTGGAATGTAGCG
RAD50 Fp	TAAGTGTGCAGAAATTGACCGAG
RAD50 Rp	GACGTACCTGCCGAAGTGTT
NBS1 Fp	AGGTGGGGAAGCTAGGTTGAT
NBS1 Rp	CACCGCCAATCCAATTTCTGC
IFI16 Fp	AGACTGAAGACTGAACCTGAAGA
IFI16 Rp	GAACCCATTGCGGCAAACATA
STING Fp	AGCATTACAACAACCTGCTACG
STING Rp	GTTGGGGTCAGCCATACTCAG
IFNβ Fp	AACTTGCTTGGATTCCTACAAAG
IFNβ Rp	TATTCAAGCCTCCCATTCAATTG
CCL5 Fp	CGCTGTCATCCTCATTGCTA
CCL5 Rp	CCAGACTTGCTGTCCCTCTC

CXCL10 Fp	CTGTACGCTGTACCTGCATCA
CXCL10 Rp	TTCTTGATGGCCTTCGATTC
Actin Fp	GGCTGTATTCCCCTCCATCG
Actin RP	CCAGTTGGTAACAATGCCATGT
Samhd1 Fp	GAGAATCGTGGTTTCCGAGAG
Samhd1 Rp	CTCCAAGGAACTTACTCCCAGA
Ifnβ Fp	CAGCTCCAAGAAAGGACGAAC
Ifnβ Rp	GGCAGTGTAACTCTTCTGCAT
Ccl5 Fp	GCTGCTTTGCCTACCTCTCC
Ccl5 Rp	TCGAGTGACAAACACGACTGC
Cxcl10 Fp	CCAAGTGCTGCCGTCATTTTC
Cxcl10 Rp	GGCTCGCAGGGATGATTTCAA
Mhc-2 Fp	TCAGTCGCAGACGGTGTTTAT
Mhc-2 Rp	GGGGGCTGGAATCTCAGGT
Cd86 Fp	TGTTTCCGTGGAGACGCAAG
Cd86 Rp	TTGAGCCTTTGTAAATGGGCA
siSAMHD1-1	CCUCGUCCGAAUCAUUGAUTT
siSAMHD1-2	GCGUAUUUGUGCUAGAGAUTT
siSAMHD1-3	GCAGAUGACUACAUAGAGATT
shSamhd1	GGATGAGGATCGTCTGGAA
siSTING	GGUCAUAUUACAUGGAUATT

Table S2. Antibodies used in this study.

Antibody	Company	Catalog number
GAPDH	Proteintech	10494-1-AP
ssDNA	Sigma-Aldrich	MAB3868
IFI16	Abcam	Ab169788
SAMHD1	Origene	TA502024
STING	Proteintech	19851-1-AP
IRF3	Proteintech	11312-1-AP
p-IRF3	Cell Signaling Technology	4947S
TBK1	Cell Signaling Technology	3013S
p-TBK1	Cell Signaling Technology	5483S
Dylight 549 Goat Anti-Mouse	Abbkin	A23310
FITC Goat Anti-Rabbit IgG	Proteintech	SA00003-2
HRP Goat Anti-Rabbit IgG	Proteintech	SA00001-2
HRP Goat Anti-Mouse IgG	Proteintech	SA00001-1
Fixable Viability Stain 700	BD Pharmingen	564997
Ms CD45 PerCP-Cy5.5	BD Pharmingen	550994
Ms CD3 APC-Cy7	BD Pharmingen	560590
PE-Cy7 Anti-mouse CD4	BD Pharmingen	552775
Flour647 Anti-mouse CD8a	BD Pharmingen	557682
Ms CD86 PE-Cy7 GL1	BD Pharmingen	560582
CD11b APC-Cy7	BD Pharmingen	557657
Ms F4/80 BV421	BD Pharmingen	565411
Ms I-A/I-E BV480	BD Pharmingen	746669

Additional Figures

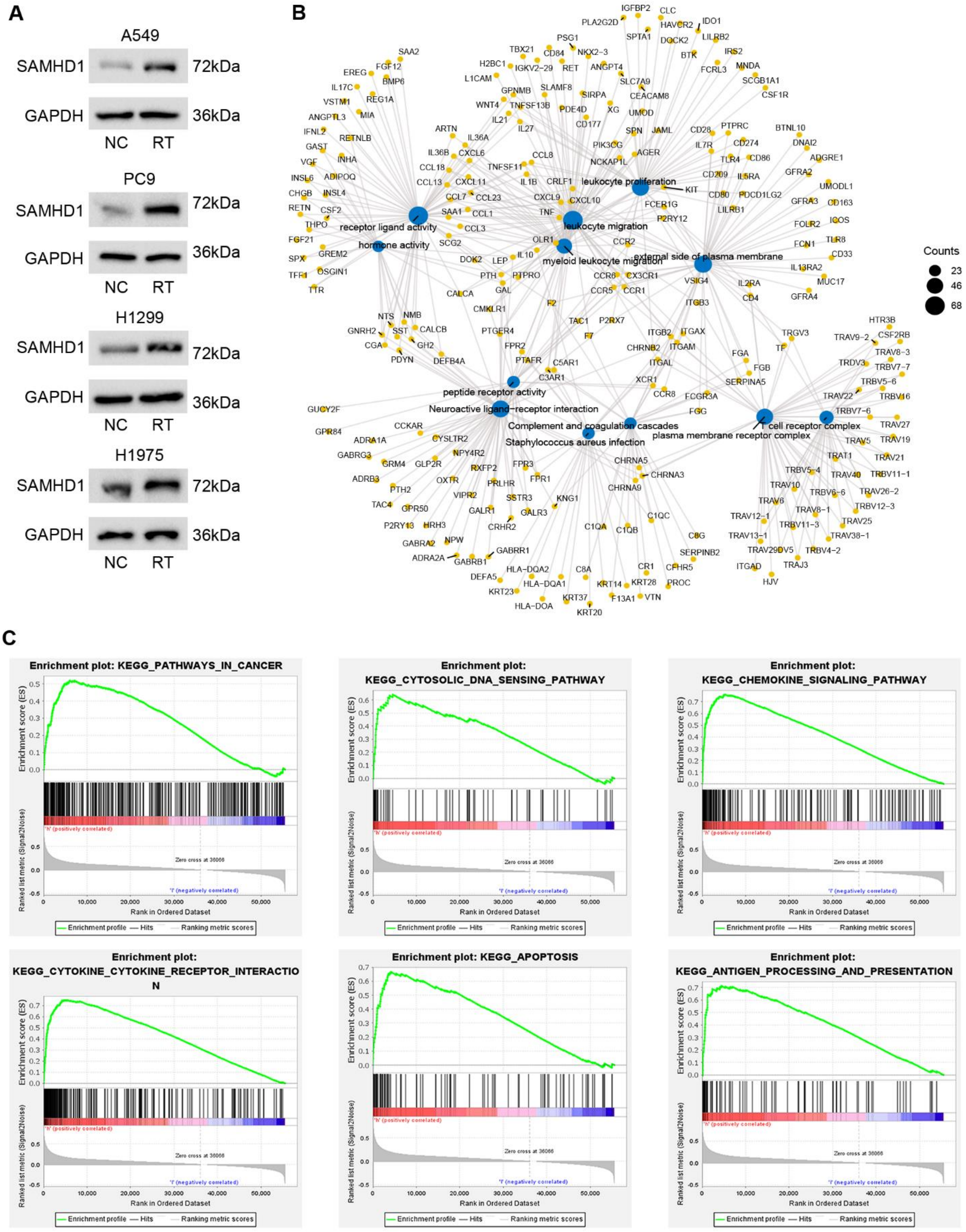

Figure S1. Functional enrichment analysis to confirm the association of SAMHD1 with immunity. (A) The SAMHD1 protein levels after radiation were detected by immunoblotting in LUAD cell lines. **(B)** GO and KEGG enrichment analysis. (C) GSEA analysis.

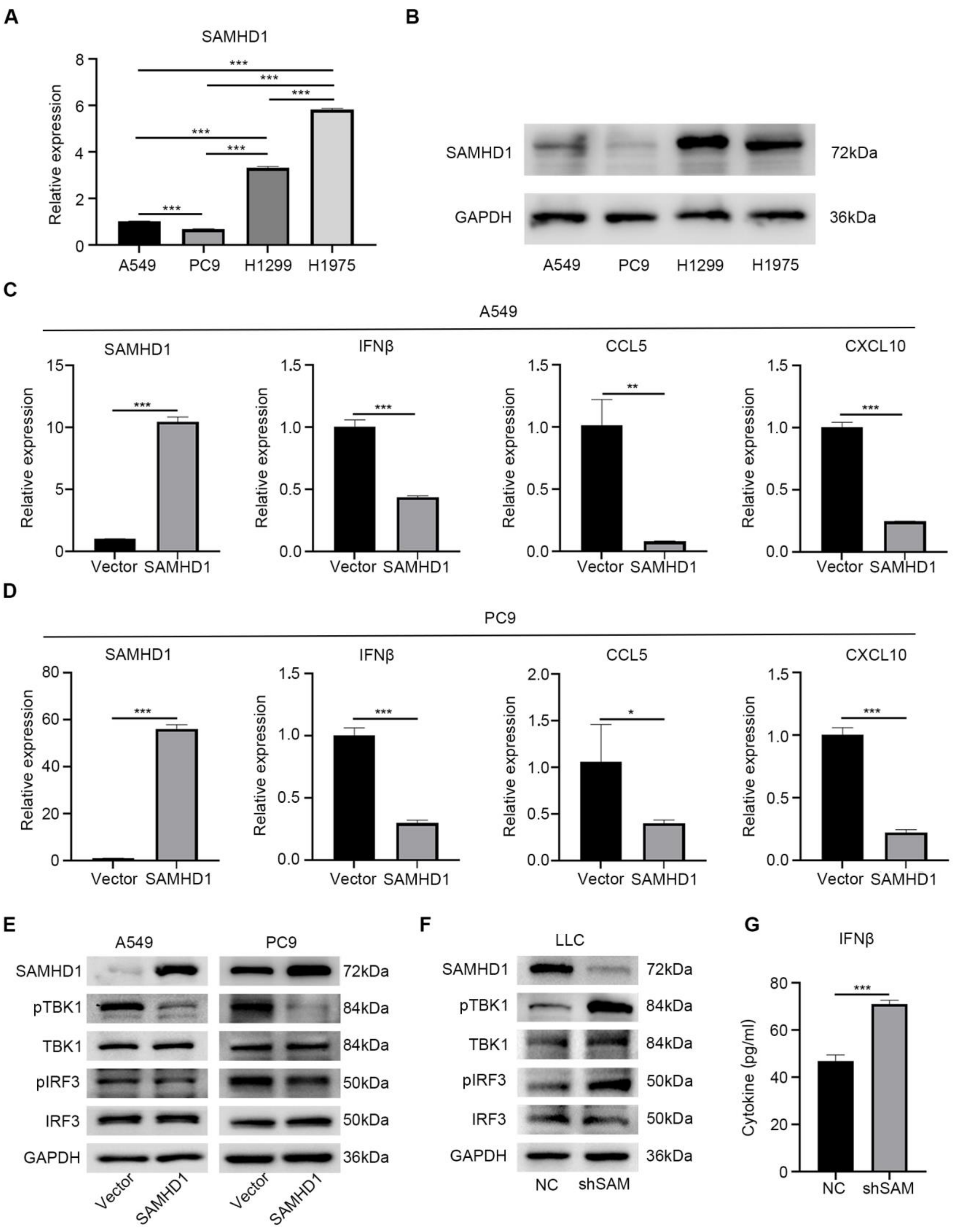

Figure S2. SAMHD1 inhibited TBK1-IRF3-IFN-I pathway in LUAD cells. (A, B) The mRNA and protein levels of SAMHD1 in LUAD cell lines. **(C, D)** The mRNA levels of IFNβ, CCL5 and CXCL10 in A549 and PC9 cells upon SAMHD1 overexpression were detected by qPCR. (E) The phosphorylation levels of TBK1 and IRF3 after SAMHD1 overexpression in A549 and PC9 cells were detected by immunoblotting. **(F)** TBK1-

IRF3 pathway protein levels in LLC cells were detected by immunoblotting. **(G)** Supernatant levels of IFNβ in LLC cells were detected by ELISA. N = 3; *, $P < 0.05$; **, $P < 0.01$; ***, $P < 0.001$.

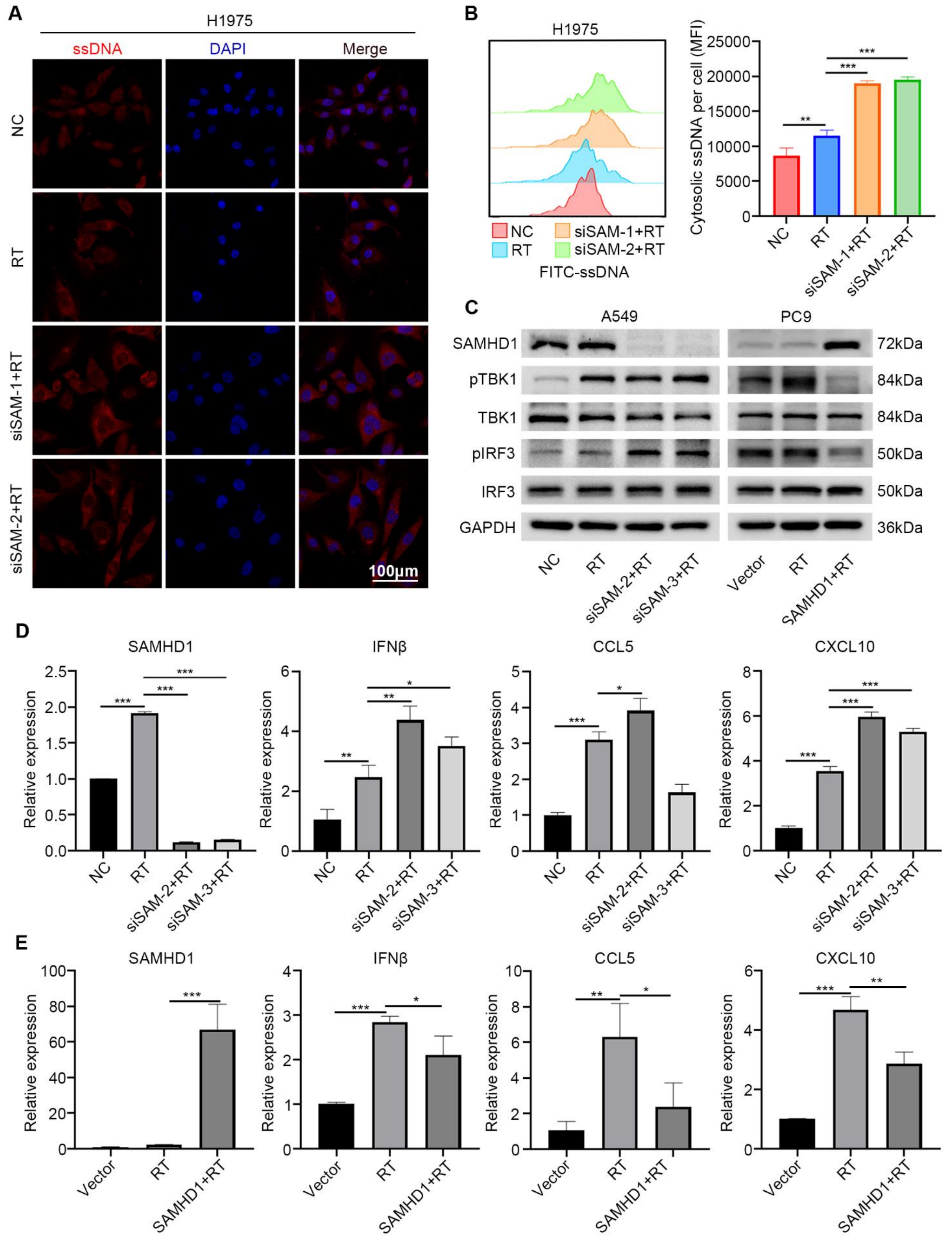

Figure S3. SAMHD1 silencing synergized with radiotherapy to induce ssDNA accumulation and activate TBK1-IRF3-IFN-I signaling. (A) Confocal images were performed to detect the accumulation of

ssDNA in cytoplasm in H1975 cells after radiation and transfecting siSAMHD1. **(B)** The accumulation of ssDNA in cytoplasm after radiation and siSAMHD1 transfection was detected by flow cytometry. The cytosolic ssDNA was evaluated by MFI. **(C)** TBK1-IRF3 pathway protein levels in A549 and PC9 cells after transfection and radiation were detected by immunoblotting.(D) The mRNA levels of SAMHD1, IFNβ, CCL5 and CXCL10 after SAMHD1 silencing and radiation in A549 cells were detected by qPCR. (E) The mRNA levels of SAMHD1, IFNβ, CCL5 and CXCL10 after SAMHD1 overexpression and radiation in PC9 cells were detected by qPCR. N = 3; *, P <0.05; **, P < 0.01; ***, P < 0.001.

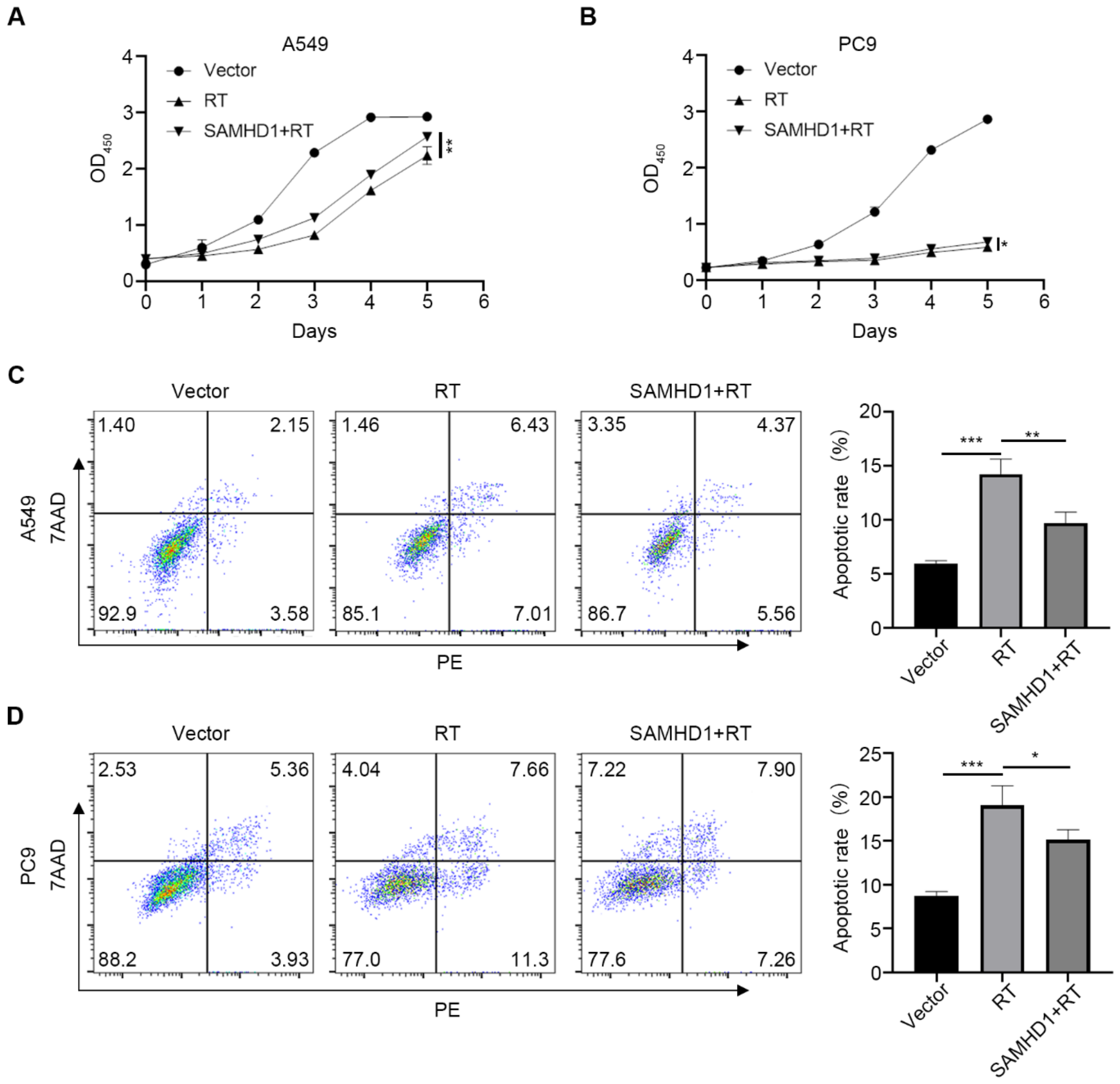

Figure S4. SAMHD1 overexpression alleviated radiation-induced inhibition of LUAD cells growth. (A, B) CCK8 assays were performed to evaluate the cell growth inhibition of SAMHD1 overexpression and radiation in A549 and PC9 cells. **(C, D)** The effects of SAMHD1 overexpression and radiation on apoptosis

were detected using flow cytometry. The statistical analyses on apoptotic rates. N = 3; *, $P < 0.05$; **, $P < 0.01$; ***, $P < 0.001$.

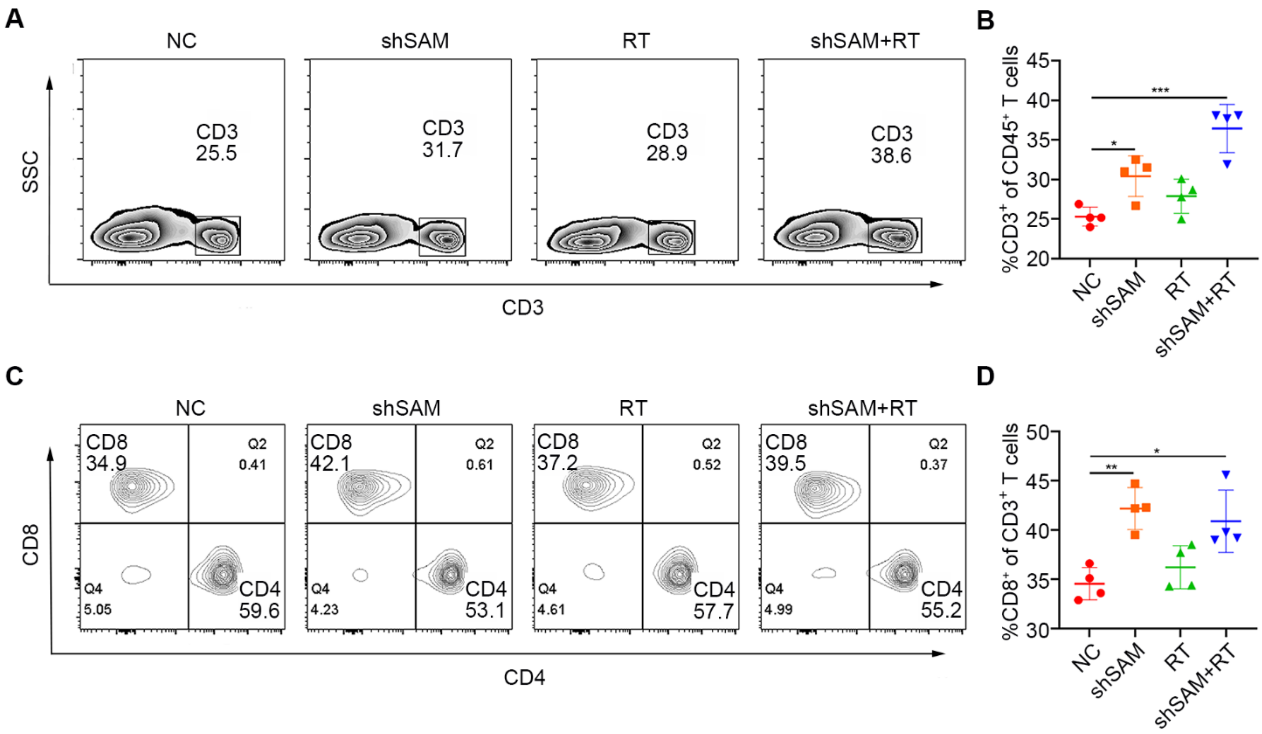

Figure S5. SAMHD1 silencing synergized with radiotherapy to regulate CD3$^+$ and CD8$^+$ T cell infiltration in spleen. (A) Representative flow cytometry of CD3$^+$ T cells in spleens. **(B)** Quantitative analysis of CD3$^+$ T cells in spleens. **(C)** Representative flow cytometry of CD4$^+$ T cells and CD8$^+$ T cells in spleens. **(D)** Quantitative analysis of CD8$^+$ T cells in spleens. N = 4; *, $P < 0.05$; **, $P < 0.01$; ***, $P < 0.001$.
